# Beyond the Dorsal Column Medial Lemniscus in Proprioception and Stroke: A White Matter Investigation

**DOI:** 10.3390/brainsci12121651

**Published:** 2022-12-02

**Authors:** Matthew J. Chilvers, Trevor A. Low, Sean P. Dukelow

**Affiliations:** 1 Department of Clinical Neuroscience, Cumming School of Medicine, University of Calgary, 3330 Hospital Drive NW, Calgary, AB T2N 4N1, Canada; 2 Hotchkiss Brain Institute, University of Calgary, 3330 Hospital Drive NW, Calgary, AB T2N 4N1, Canada

**Keywords:** proprioception, stroke, white matter, robotics, sensation, neuroimaging, neuroanatomy

## Abstract

Proprioceptive deficits are common following stroke, yet the white matter involved in proprioception is poorly understood. Evidence suggests that multiple cortical regions are involved in proprioception, each connected by major white matter tracts, namely: Superior Longitudinal Fasciculus (branches I, II and III), Arcuate Fasciculus and Middle Longitudinal Fasciculus (SLF I, SLF II, SLF III, AF and MdLF respectively). However, direct evidence on the involvement of these tracts in proprioception is lacking. Diffusion imaging was used to investigate the proprioceptive role of the SLF I, SLF II, SLF III, AF and MdLF in 26 participants with stroke, and seven control participants without stroke. Proprioception was assessed using a robotic Arm Position Matching (APM) task, performed in a Kinarm Exoskeleton robotic device. Lesions impacting each tract resulted in worse APM task performance. Lower Fractional Anisotropy (FA) was also associated with poorer APM task performance for the SLF II, III, AF and MdLF. Finally, connectivity data surrounding the cortical regions connected by each tract accurately predicted APM task impairments post-stroke. This study highlights the importance of major cortico–cortical white matter tracts, particularly the SLF III and AF, for accurate proprioception after stroke. It advances our understanding of the white matter tracts responsible for proprioception.

## 1. Introduction

Proprioception is our sense of limb position and limb movement, that arises from within the muscles and joints and can act independently from vision [1,2,3]. Proprioception makes up an important component of motor control, allowing us to move within, and interact with our surrounding environment. Following stroke, proprioceptive deficits are common, occurring in approximately 50% of cases [4,5]. These deficits are often associated with negative consequences after stroke, such as longer hospital stays and reduced ability to perform activities of daily living [5,6,7,8,9]. Despite the clear clinical importance of proprioception, the underlying proprioceptive neuroanatomy, specifically the white matter, remains relatively poorly understood.

Historically, proprioception has been viewed as a function of the ascending dorsal column medial lemniscus (DCML) and spinocerebellar pathways, with little recognition given to other white matter tracts beyond these. As such, the DCML is considered the primary white matter pathway for proprioception [10]. While the DCML is fundamental to the understanding of proprioceptive anatomy, it has been suggested that lesser discussed cortico–cortical white matter pathways may also have a role in proprioception [11,12,13].

Over the past two decades, a substantial amount of research has gone into better understanding the neuroanatomy responsible for processing a variety of different proprioceptive stimuli in humans (passive arm movement, muscle tendon vibration, limb position matching). These studies, however, have predominantly focused on the grey matter correlates of proprioception, identifying a wide network of cortical and subcortical structures that are involved in proprioception. Cortical grey matter structures involved include parietal regions (S1, supramarginal gyrus, angular gyrus, superior parietal lobe, parietal operculum), frontal and prefrontal regions (supplementary motor area, dorsal premotor cortex, ventral premotor cortex, motor cortex), temporal regions (superior temporal gyrus) and insula [14,15,16,17,18,19,20,21,22,23,24].

**Figure 1:**
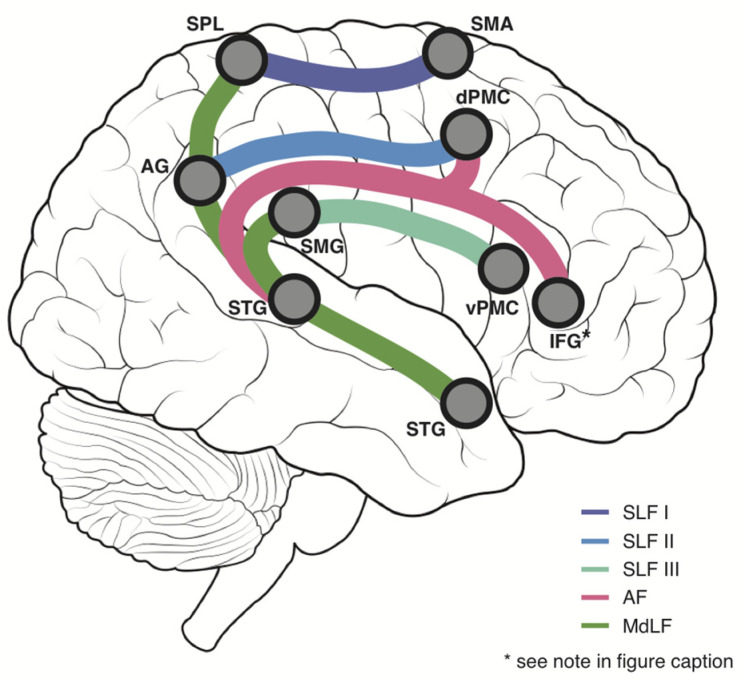
White Matter Schematic—A simplified schematic displaying the major white matter tracts of interest in the current study and their principal connections. Purple = Superior Longitudinal Fasciculus I (SLF I), Blue = Superior Longitudinal Fasciculus II (SLF II), Cyan = Superior Longitudinal Fasciculus III (SLF III), Pink = Arcuate Fasciculus (AF), Green = Middle Longitudinal Fasciculus, AG = Angular gyrus, dPMC = Dorsal Premotor Cortex, IFG = Inferior Frontal Gyrus, SMA = Supplementary Motor Area, SMG = Supramarginal gyrus, SPL = Superior Parietal Lobule, STG = Superior Temporal Gyrus, vPMC = Ventral Premotor Cortex. * Given that it forms a major connection of the AF but is overwhelmingly involved in language functions, the IFG is shown for illustrative purposes, but does not form part of the proprioceptive network connectivity analysis in the present study.

Many of the aforementioned regions are connected by three large white matter tracts and their subdivisions: the superior longitudinal fasciculus (SLF I, SLF II and SLF III), the arcuate fasciculus (AF) and the middle longitudinal fasciculus (MdLF) [12]. Figure 1 provides a simple schematic of these white matter connections.

### 1.1. Superior Longitudinal Fasciculus

The superior longitudinal fasciculus (SLF) is comprised of three branches, connecting the parietal lobe with frontal regions. Post-mortem dissections in humans demonstrate that the angular gyrus and supramarginal gyrus connect with the frontal lobe via the SLF [25]. Investigations of the SLF I, using both diffusion spectrum imaging (DSI) in non-human primates and diffusion tensor imaging (DTI) in humans, have shown that it courses from the superior parietal lobe towards the supplementary motor area (SMA) and medial premotor regions [26,27]. The SLF II has shown to extend more laterally than the SLF I, connecting parts of the inferior parietal lobe, namely the angular gyrus [25], with dorsal aspects of the premotor cortex (dPMC) and prefrontal cortex [26,27]. Finally, the SLF III lies more ventral than the SLF II, connecting regions of the inferior parietal lobe corresponding to the supramarginal gyrus [25], with the ventral premotor cortex (vPMC) and prefrontal cortex [26,27,28].

### 1.2. Arcuate Fasciculus

In historic post-mortem dissection work, the AF and SLF were originally described as a single fibre pathway, not distinguished from one another [29,30,31,32]. Only more recently have the AF and SLF been described as separate white matter pathways [26,28,33,34,35,36,37], with the AF generally being coined a partition of the SLF [38]. Histological and diffusion imaging techniques have allowed greater understanding the trajectories of these white matter bundles. While descriptions of the AF often focus on the connection between Wernicke’s and Broca’s area [39,40], the AF has also been shown to connect the posterior superior temporal gyrus with more dorsal aspects of the premotor cortex, inferior parietal lobule, in addition to its classical IFG/Broca’s connection [26,41,42,43,44].

The AF has predominantly been described as the major white matter pathway sub-serving language functions, particularly in the left hemisphere [35,38,45]. The left AF may also support skilled voluntary movement, with lesions to the left AF inducing ideomotor apraxia [36,46]. Right AF function is less well documented, with some suggestions of a visuospatial role and involvement with spatial neglect [47,48].

### 1.3. Middle Longitudinal Fasciculus

The middle longitudinal fasciculus, originally described in the macaque using autoradiography and, later, diffusion spectrum imaging (DSI) [12,26,33,49], connects the inferior parietal and temporal lobes. Human DTI evidence has supported this, showing the MdLF to connect the superior temporal gyrus with the angular gyrus [50,51]. DTI studies in humans have also shown the MdLF to extend beyond the angular gyrus to the supramarginal gyrus, superior parietal lobe and parieto-occipital regions [43,52,53,54,55], something also supported by DSI work in the macaque [56]. While less is known about the functional aspects of the MdLF, given the regions it interconnects, it is suggested to support language in the dominant hemisphere, and visuospatial and attentional functions in the non-dominant hemisphere [12,33,50,51,52,53]. The close relationship between attentional and proprioceptive deficits [57,58,59] makes the MdLF another candidate tract for the current investigation.

### 1.4. Aims and Hypothesis

Although the above white matter tracts connect cortical regions of the brain that are reported to subserve proprioception and bodily awareness, direct evidence supporting the SLF and/or AF in proprioception is limited [11,16,17,60]. In the current study, diffusion imaging was used to investigate how structural properties of these specific white matter tracts might relate to performance on an Arm Position Matching (APM) task in stroke and control participants.

It was hypothesised that participants with lesions impacting any of the SLF I, SLF II, SLF III, AF and MdLF would perform significantly worse than stroke participants without lesions impacting these tracts, as well as controls, on a robotic Arm Position Matching (APM) task. Further, it was hypothesised that Fractional Anisotropy (FA) of each of these tracts would be lower in participants with proprioceptive impairments and lower FA would be associated with poorer performance on the APM task.

## 2. Materials and Methods

### 2.1. Participant Recruitment

A total of 33 individuals were recruited to participate in this study (26 participants with stroke and seven control participants without stroke). Stroke participants were recruited from the Foothills Medical Centre, Calgary, Alberta, Canada. Inclusion criteria for the current study were as follows: First time clinical presentation of ischaemic or haemorrhagic stroke, no contraindications to MRI, no previous diagnosis of other neurological disorders, 18 years of age or older, ability to understand and follow instructions for robotic and clinical assessments. Exclusion criteria were evidence of apraxia (determined by a clinical screen for apraxia) [61], cerebellar or brainstem strokes and orthopaedic injuries to the upper extremities. All participants gave written informed consent. This study was approved by the Conjoint Health Research Ethics Board at the University of Calgary.

### 2.2. MRI Acquisition

MRIs were acquired for all stroke participants approximately 4 weeks post-stroke (mean 27.1 ± 11.2 days). This timeframe was chosen to allow for sufficient resolution of edema across participants [62,63], and the effect of the stroke to be apparent on diffusion metrics [64,65,66]. Imaging was acquired using a 3T Discovery MR 750 scanner (General Electric, Boston, United States). Acquisition sequences included diffusion weighted imaging (DWI), T1-weighted imaging and fluid attenuated inversion recovery imaging (FLAIR).

#### 2.2.1. Diffusion-Weighted Imaging

The DWI scans were acquired using the following parameters: TR = 8000 ms, TE = 61 ms, matrix size = 110 × 110, axial slices = 80, voxel size = 2.2 mm^3^, diffusion directions = 90, b-value = 1000 s mm^−2^, and acceleration factor in phase encoding direction = 2, SENSE (ASSET). In addition, nine volumes were acquired with reverse phase encoding. Six volumes were acquired with a b-value of 1000 s mm^−2^ and the remaining three volumes without diffusion weighting.

#### 2.2.2. T1-Weighted Imaging

The T1 images were acquired with the following parameters: TR = 6.656 ms, TE = 2.9 ms, inversion time = 650 ms, flip angle = 10°, matrix size = 256 × 256 × 192, voxel size = 1 mm^3^, and acceleration factor in phase encoding direction = 2, GRAPPA (ARC).

#### 2.2.3. FLAIR Imaging

The FLAIR images were acquired with the following parameters: TR = 4200 ms, TE = 75 ms, inversion time = 1367 ms, slice thickness = 1.5 mm, matrix size = 256 × 150 × 130, GRAPPA (ARC).

### 2.3. Image Pre-Processing

Images were pre-processed utilising common toolboxes in FSL [67,68]. Estimation of susceptibility-induced distortions was performed using the TOPUP tool [69,70] with the distortion field map being used in the subsequent step to correct these distortions, as well as eddy currents and head motion. This correction was performed using the Eddy tool [71]. In order to calculate FA and MD for each tract, tensors were fit to each voxel using the DTIfit tool. To generate the appropriate transformations to run tractography in native space, the diffusion images were registered to standard MNI space for each participant using the linear (FLIRT) and non-linear (FNIRT) transformation tools in FSL [72,73]. First, each diffusion image was registered to the participants T1-weighted image using FLIRT. Next, a non-linear approach (FNIRT) was used to register the T1 image to MNI152 space. Finally, the diffusion to T1 and T1 to MNI transforms was combined to allow the transformation from diffusion space to MNI space to occur. This transformation was also inverted to allow the MNI to diffusion transformations. Registrations were checked for accuracy by ensuring the alignment of key landmarks such as the lateral ventricles and the overall brain outline. This was done by overlaying the MNI brain on the registered brain for each participant.

### 2.4. Probabilistic Tractography of SLF I, II, III, AF and MdLF

In order to model crossing fibres and calculate estimates of diffusion parameters at each voxel, the BedpostX (Bayesian Estimation of Diffusion Parameters Obtained using Sampling Techniques) toolbox in FSL was used, accounting for two fibres per voxel. The output from BedpostX was then used to perform probabilistic tractography. Tractography was performed utilising the freely available toolbox in FSL, called XTRACT [74], which provides a standardised approach to performing tractography for well identified white matter tracts. For each participant, tractography was performed in native (diffusion) space and utilised the following tracking parameters, default to XTRACT: (step length = 0.5 mm, curvature threshold = ±80°, maximum number of steps = 2000). A threshold of 1% was then applied to the probabilistic tracts, and binary masks of each tract created. Fractional Anisotropy (FA), mean diffusivity (MD) and tract volume of each tract was then calculated.

### 2.5. Proprioceptive Network Connectivity

In addition to the FA and MD of the SLF, AF and MdLF, the structural connectivity (number of streamlines) surrounding a network matrix of cortical regions, known to be connected by these tracts and implicated in human proprioception, was assessed. Regions in this network included S1, supramarginal gyrus, angular gyrus, superior parietal lobe, supplementary motor area, dorsal premotor cortex, ventral premotor cortex and superior temporal gyrus.

Binary masks were made for each region of interest, using the Oxford-Harvard Cortical Structural atlas, distributed though the FSL package—https://fsl.fmrib.ox.ac.uk/fsl/fslwiki/Atlases (accessed on 23 January 2019). These masks were then registered to each participant’s diffusion space using the transformations outlined above. Probabilistic tractography was then performed using each region as both a seed and target. The same parameters, default to XTRACT, were utilised [74]: (step length = 0.5 mm, curvature threshold = ±80°, maximum number of steps = 2000, number of samples = 5000). Tractography was corrected for distance, using the distance correction setting in FSL. For each participant, a white matter mask was also used to constrain the fibre tracking. Because seed volume can impact the number of streamlines generated, the number of streamlines between each region pair was normalized by dividing the number of streamlines from each ROI by the volume of the seed ROI.

As such, the number of streamlines reaching each region (ROI_a_) was quantified, when each other region (ROI_b_) was used as a seed. For each pair of regions, the average number of streamlines connecting the two regions was calculated. These connectivity values were then used to see how the connectivity between each region of this network could predict impairment status on the APM task.

### 2.6. Lesion Marking and Registration

For participants in the stroke group, lesions were marked on the original FLAIR image. The markings were then registered to both the participants diffusion scan, as well as to MNI space. Lesion markings were registered from FLAIR to diffusion space by way of linear transformation, utilising the FLIRT tool in FSL.

In order to register the lesion markings from FLAIR to MNI space, the FLAIR image was first registered to the T1 image using a linear transformation. The T1 image was then linearly transformed to MNI space, and the linear transformation from this step used to transform the T1 image to MNI space by way of a non-linear transformation. Finally, these two transformation steps were combined to create a FLAIR to MNI transformation. All registrations were visually inspected and compared to the original markings for accuracy.

### 2.7. Control Tracts and Lesion Overlap

In order to produce template white matter tracts, typical for controls, overlays of each respective tract were created from the control participants tracts, registered to MNI space. Overlays were created in MRIcron [75]. Binary masks of the overlaid control tracts were created by taking voxels that were only present in the tracts of at least six of the seven control participants (86%).

These template tracts were then used to determine whether participants in the stroke group had lesions impacting each respective tract. To determine this, the template tracts from the controls were multiplied by the binary MNI lesion markings for each participant (described above) to give only the voxels/volume present in both masks. If a participant had lesioned voxels overlapping with a given tract, they were assigned to the lesion group for that tract (e.g., SLF I lesions). *It is worth noting that a single participant could belong to multiple lesion groups based on this classification*. Consequently, differences in APM Task Scores were assessed for participants in the lesioned (percent damage > 0%) and non-lesioned (percent damage = 0%) groups for each white matter tract, in addition to controls (i.e., SLF I lesions vs. No-SLF I lesions vs. Controls).

### 2.8. Robotic Assessments

**Figure 2:**
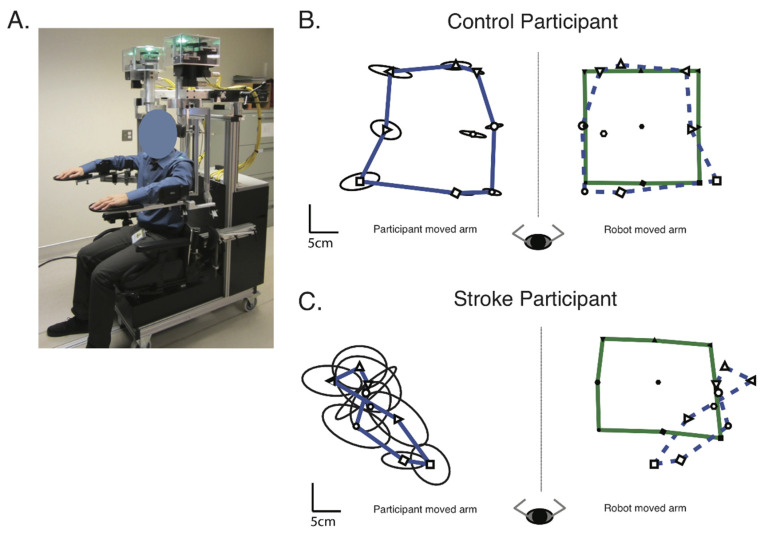
Methods—(**A**) The Kinarm Exoskeleton robotic device used to perform the Arm Position Matching task. (**B**) Exemplar of a control participant’s performance on the Arm Position Matching task. The robot moved the control participant’s dominant (right) arm. The mean position the robot moved the participant’s arm to for each target, across the six trials per target, are indicated by the black, filled symbols. The green outline connects the mean position of the outer eight targets of the robot moved arm. The participant then matched with their non-dominant (left) arm, with the mean matched positions indicated by the black, open symbols. The blue line connects the mean matched position of the outer eight targets, for the participants actively moved arm. For illustrative purposes, the mean position of the matched targets and their outline (blue dashed line) have been reflected across the midline onto the robot moved positions. Ellipses around each target indicate one standard deviation of variability in the matched target positions. (**C**) Exemplar of a stroke participant’s performance on the Arm Position Matching task. Format the same as (**B**), except the robot moved the participant’s affected (right) arm and they matched with their non-affected (left) arm.

All participants completed a robotic APM task, to assess arm position sense. They performed the APM task 22.6 ± 11.9 days (mean ± std) post-stroke and within 4.5 ± 16.2 days (mean ± std) of imaging. The APM task was conducted using a Kinarm Exoskeleton Lab (Kinarm, Kingston, ON, Canada) (Figure 2a). The APM task has previously been validated in stroke [76] and has been studied extensively in relation to proprioceptive deficits, recovery and neuroanatomy [5,17,18,57,59,76].

To perform the APM task, participants sat in the wheelchair base of the robot exoskeleton. The arm troughs on the robotic device were then adjusted to fit each participant, using the adjustable linkages. As such, the participant’s arms were supported against gravity in the horizontal plane. The robotic device was then wheeled into the virtual reality environment where the participants performed the task. All participants had vision of their upper limb occluded by an opaque screen and a bib placed over the shoulders. For the entirety of the robotic assessment, participants were under the close supervision of the robot operator, who ensured they performed the APM task to the best of their ability. No participants complained of dizziness during the assessment.

The APM task consisted of nine spatial targets, oriented in a square, with eight outer targets surrounding a central target (Figure 2b). The central target was positioned at the point where the elbow was flexed at 90° and the shoulder horizontally adducted to 30°. The eight outer targets were each spaced 10 cm apart.

First, the robot moved one of the participant’s arms (passive arm) to one of the nine targets. For stroke participants, the passive arm was their affected arm. For control participants, the passive arm was their dominant arm. The passive arm was moved with a bell-shaped velocity profile, with a peak speed of 0.3–0.5 m/s. Once the robot had finished moving the passive arm to its location, participants were instructed to mirror-match the position of the passive arm, with their other arm (active arm). Once the participant felt they were in a matched position, they seudorand to the robot operator that they had matched, and the operator cued the robot to the next trial. Each of the nine targets was assessed in a seudorandomized order within a single block. There were six blocks in the APM task, totalling 54 trials.

#### APM Task Score

Performance on the APM task was quantified by a global measure called an APM Task Score, which combined scores from a number of spatial parameters into a single performance metric and compared performance to a normative dataset. First, parameter scores were calculated: absolute error, variability, contraction/expansion and shift. Briefly, absolute error refers to the distance in error between the robot and participant moved arms. Variability refers to the trial-by-trial variability in matched position. Contraction/expansion refers to the perception of the workspace being shrunken or enlarged. Finally shift describes a perceived systematic shift in the workspace in the x and y directions. All parameters were calculated for both the x and y directions. For further details on how each parameter was calculated, see Supplemental Methods SA and https://kinarm.com/download/kst-summary-analysis-version-3-9/ (accessed on 25 March 2021).

Once these parameters were calculated in their raw unit form, the next step was to convert each parameter score into a z-score, based on a large normative dataset comprising of 2227 APM task exams, from 799 healthy controls with no history of neurological disorders (363 males, 436 females, ages 18–93). As such, these z-scores account for each participant’s age, sex and handedness. The next step was to convert each z-score to a zeta score, such that for each parameter, the best possible score was indicated by a value of zero. The final step in calculating the APM Task Score was to calculate the root-sum-square (RSS) distance of the parameter z and zeta scores and then transforming this RSS into a z-score, by Box-Cox transform, again accounting for the normative dataset. Finally, these z-scores were transformed into zeta-scores using the same transform as the parameter z-scores [77,78].

A key feature of the APM Task Score, Is that 95% of healthy controls have an APM Task Score < 1.96. As such, a score > or equal to 1.96 was used to indicate whether a participant had impairments on the APM Task.

Robotic analysis was performed in Dexterit-E version 3.9 (Kinarm Standard Tests, BKIN Technologies Ltd., Kingston, ON, Canada). A detailed account of this process, the contribution of each parameter to the APM Task Score and the calculations performed are freely available: https://kinarm.com/download/kst-summary-analysis-version-3-9/ (accessed 25 March 2021). Figure 2b,c provide exemplars of a control and stroke participant’s performance on the APM task, respectively.

### 2.9. Clinical Assessment

In addition to the APM task, participants also performed a number of clinical assessments, to provide a more detailed description of the clinical presentation of the study participants. To assess motor impairments contralesionally and ipsilesionally, the Chedoke McMaster Stroke Assessment (CMSA) was performed on each arm [79]. Scores less than 7 indicated motor impairments on either arm. To detect the presence of hemispatial neglect, the Behavioural Inattention Test (BIT) was conducted [80]. Scores less than 130 indicated the presence of hemispatial neglect. Participants also performed the Functional Independence Measure (FIM) [81], Montreal Cognitive Assessment (MoCA) [82], and the Edinburgh Handedness Inventory [83] to assess the ability to perform activities of daily living, cognitive impairment and handedness, respectively.

### 2.10. Statistical Analysis

Statistical analysis was performed in Matlab 2019 (Natick, MA, USA), SPSS Version 28 and Scikit toolbox in Python [84].

#### 2.10.1. Analysis of Variance

To assess differences in APM Task Scores (dependent variable) between those with and without lesions impacting each of the tracts and the seven controls (independent variable), one-way ANOVAs were performed. Of the 26 stroke participants, the number of participants with lesions to each tract were as follows: SLF I = 3, SLF II = 6, SLF III = 8, AF = 20, MdLF = 9. In cases where the assumption of equal variance between groups was violated, Welch’s tests were performed, with Games–Howell post hoc tests performed. In instances where equal variance was met, Sidak corrections were used to correct for multiple comparisons.

One-way ANOVAs were also performed to infer differences in FA, MD and tract volume (dependent variable) between the lesioned and non-lesioned hemispheres of those with and without impairments on the APM task (APM Task Score > 1.96) and the left and right hemispheres of control participants (independent variable).

#### 2.10.2. Linear Regression

In order to evaluate the relationship with APM Task Scores (dependent variable) and FA, MD and tract volume for each tract (independent variable), linear regressions were conducted. An alpha level of 0.05 was used to infer significance. Benjamini–Hochberg corrections were used to correct for multiple comparisons across the five tracts, with a false discovery rate of 5%.

#### 2.10.3. Logistic Regression

To determine whether different diffusion metrics could predict if an individual was impaired on the APM task (Task Score > 1.96), three logistic regression models were trained. Each model utilized an independent feature selection method, in order to try to optimize the model in a stepwise manner and identify the features most important to classify impairment status on the APM task (dependent variable).

The first model trained contained all possible features (All Features model), including the FA and MD values for the SLF I, SLF II, SLF III, AF and MdLF, in addition to the connectivity values (number of streamlines) between each pair of ROIs in the connectivity matrix, described in Section 2.5 (38 features total). The second model utilized a Univariate Feature Selection method to reduce the multicollinearity in the dataset and aimed to improve classification accuracy (Univariate Feature Selection model). To identify the collinear features, Spearman’s rank correlations were conducted between each pair of features. A feature was removed if it had an r > 0.7 and *p* < 0.05 with another feature, in which the feature with the greatest mean coefficient with all other features was removed [85]. Furthermore, due to the particular interest of the current study in parietal–frontal, temporal–frontal and temporal–parietal connectivity, connectivity values between vPMC-dPMC, vPMC-SMA and dPMC-SMA were also removed. As such, the Univariate Feature Selection models contained 26 features total (7 FA/MD metrics, 19 connectivity values). From the Univariate Feature Selection model, Recursive Feature Elimination was then used to identify the optimal number of features that maximized prediction accuracy and find the anatomical features which were most informative for accurate classification of APM task impairments. Finally, a model was trained with the optimal features identified in this step (Recursive Feature Elimination model), with the aim of being the most accurate predictor of APM impairment status. The Recursive Feature Elimination model contained 13 features (4 FA/MD metrics, 9 connectivity values).

Before training each model, the dataset was converted to standardized scores, with a mean of zero and standard deviation of one. Performance of each of the three models was evaluated using 10-fold cross validation, selected due to its bias and variance properties with smaller sample sizes [85] and given it has performance measures similar to Leave-One-Out cross validation [86]. For each model, the accuracy, precision, recall, f1-score and the most important features were all calculated and identified respectively. The most important features were determined by calculating the mean coefficient for each variable across the 10-folds.

## 3. Results

### 3.1. Participant Demographics

Participant demographics are presented in Table 1. Stroke participants (16 male, 10 female) were, on average, 63.0 ± 13.4 years of age. Control participants (3 male, 4 female) were, on average, 62.1 ± 6.2 years of age. Nineteen stroke participants had right hemisphere lesions and left affected arms. The remaining seven stroke participants had left hemisphere lesions and right affected arms. Proprioceptive impairments, as per the APM task, were present in 15 of the 26 stroke participants (57%). As per the TLT, they were present in 11 of the 26 stroke participants. Contralesional motor impairments, as per the CMSA, were apparent in 21 of the 26 participants. Ipsilesional motor impairments were rare, and mild when present, occurring in only four participants, as per the CMSA. Within the sample, two participants had hemispatial neglect, as per the BIT (scores <130). Both participants also were impaired on the APM task.

### 3.2. Lesion and Tract Information

**Figure 3:**
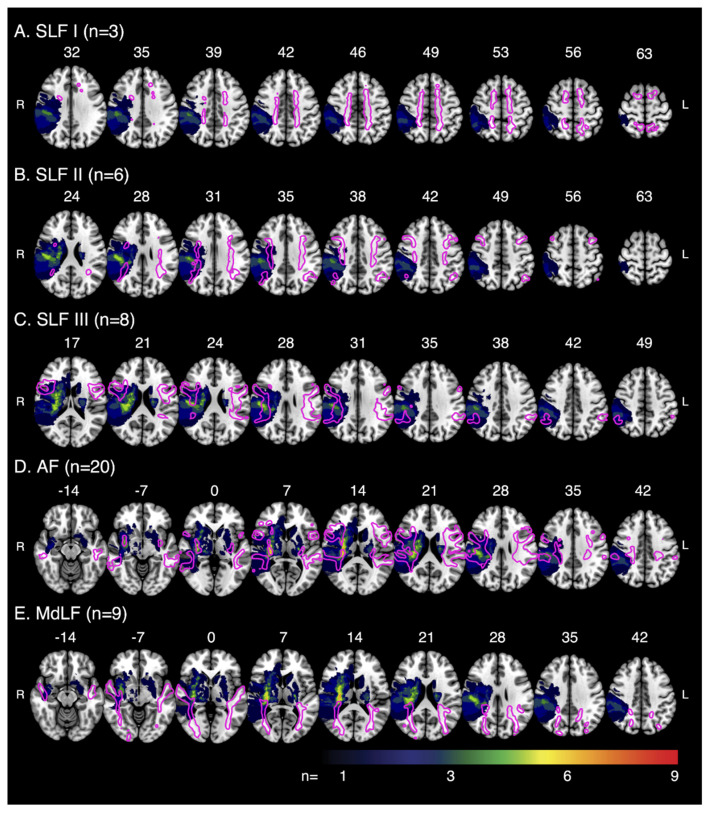
Tract–Lesion Overlaps—Multislice images overlaying the template white matter tracts, typical for control participants, on the lesion overlap for stroke participants. Images depict the amount of overlap between the white matter tracts and lesions for (**A**) SLF I (n with lesions = 3), (**B**) SLF II (n with lesions = 6), (**C**) SLF III (n with lesions = 8), (**D**) AF (n with lesions = 20) and (**E**) MdLF (n with lesions = 9). Images displayed in radiologic convention, with the right hemisphere on the left. Tracts and lesions are normalised to Montreal Neurological Institute 152 space. SLF = Superior Longitudinal Fasciculus, AF = Arcuate Fasciculus, MdLF = Middle Longitudinal Fasciculus.

Figure 3 displays the overlap between the template white matter tracts, typical for controls (see Section 2.7) and lesion map for all participants. Across all stroke participants, mean lesion volume was 11.97 cc ± 16.60 cc. MD and tract volumes are presented in Supplemental Results SA. Across the sample, participants had lesions impacting varying combinations of white matter tracts. Few participants had lesions impacting the SLF I (Supplemental Results SB). There were also six participants with lesions that did not impact any of the respective tracts. FA for each group is presented later and in Supplemental Results SC.

### 3.3. Differences in APM Task Scores with White Matter Tract Lesions of the SLF I, SLF II, SLF III, AF and MdLF

The first stage of analysis compared differences in APM Task Scores between stroke participants with and without lesions to each of the SLF I, SLF II, SLF III, AF and MdLF, as well as controls. Two of the six participants without lesions to any of the respective tracts were impaired on the APM task (Supplemental Results SB).

#### 3.3.1. SLF I

**Figure 4:**
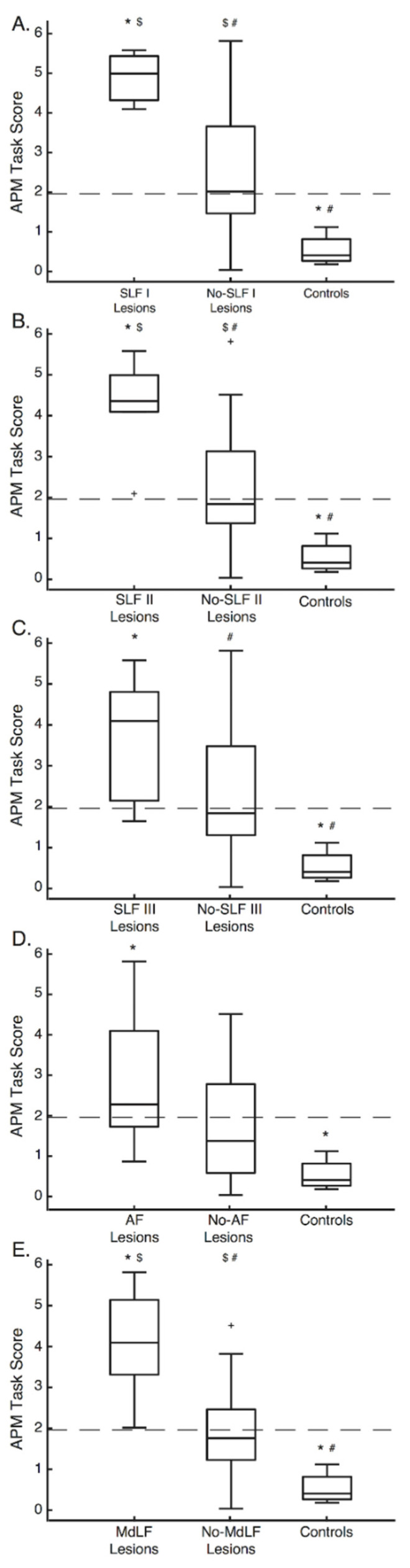
Arm Position Matching Task Score Box-plots—(**A**) Arm Position Matching Task Scores for participants with lesions to the SLF I (SLF I Lesions), participants without lesions to SLF I (No-SLF I Lesions) and control participants. Dashed line at a Task Score of 1.96 represents the upper range of normal performance. For each box, the centre line is the median and the bottom and top lines represent the 25th and 75th percentile, respectively. Whiskers extend to the highest and lowest data points, within 1.5 times the interquartile range from the top or bottom of the box. + indicate data points outside of this range and signify outliers. *, $ and # indicate groups with significant differences between them. (**B**–**E**) Same format as (**A**), except for the SLF II, SLF III, AF and MdLF respectively. SLF = Superior Longitudinal Fasciculus, AF = Arcuate Fasciculus, MdLF = Middle Longitudinal Fasciculus, APM = Arm Position Matching.

For the SLF I, the ANOVA revealed significant differences in APM Task Scores between the stroke groups and controls (F(2, 5.66) = 50.62, *p* = 0.00025) (Figure 4a). Post hoc testing revealed Task Scores were higher/worse for participants with SLF I lesions compared to both those without SLF I lesions (*p* = 0.016) and controls (*p* = 0.011). Task Scores were also higher/worse for participants without SLF I lesions compared to controls (*p* = 0.16 × 10^−4^).

#### 3.3.2. SLF II

For the SLF II, significant differences in APM Task Scores were observed (F(2,30) = 14.31, *p* = 0.43 × 10^−4^) (Figure 4b). Task Scores were higher/worse for participants with SLF II lesions compared to both those without SLF II lesions (*p* = 0.0046) and controls (*p* = 0.26 × 10^−4^). Task Scores were also higher/worse for participants without SLF II lesions compared to controls (*p* = 0.013).

#### 3.3.3. SLF III

For the SLF III, significant differences in APM Task Scores were observed (F(2,14.90) = 23.90, *p* = 0.22 × 10^−4^) (Figure 4c). Task Scores were higher/worse for participants with SLF III lesions than controls (*p* = 0.0011). Task Scores were also higher/worse for participants without SLF III lesions compared to controls (*p* = 0.00049).

#### 3.3.4. AF

For the AF, significant differences in APM Task Scores were observed (F(2,11.51) = 21.56, *p* = 0.00013) (Figure 4d). Task Scores were higher/worse for participants with AF lesions compared to controls (*p* = 0.30 × 10^−5^). No other comparisons were significantly different.

#### 3.3.5. MdLF

For the MdLF, significant differences in APM Task Scores were observed (F(2,30) = 21.35, *p* = 0.20 × 10^−5^) (Figure 4e). Task Scores were higher/worse for participants with MdLF lesions compared to both those without MdLF lesions (*p* = 0.00015) and controls (*p* = 0.20 × 10^−5^). Task Scores were also higher/worse for those without MdLF lesions, compared to controls (*p* = 0.027).

### 3.4. Differences in FA between Those Impaired and Those Unimpaired on the APM Task

**Figure 5:**
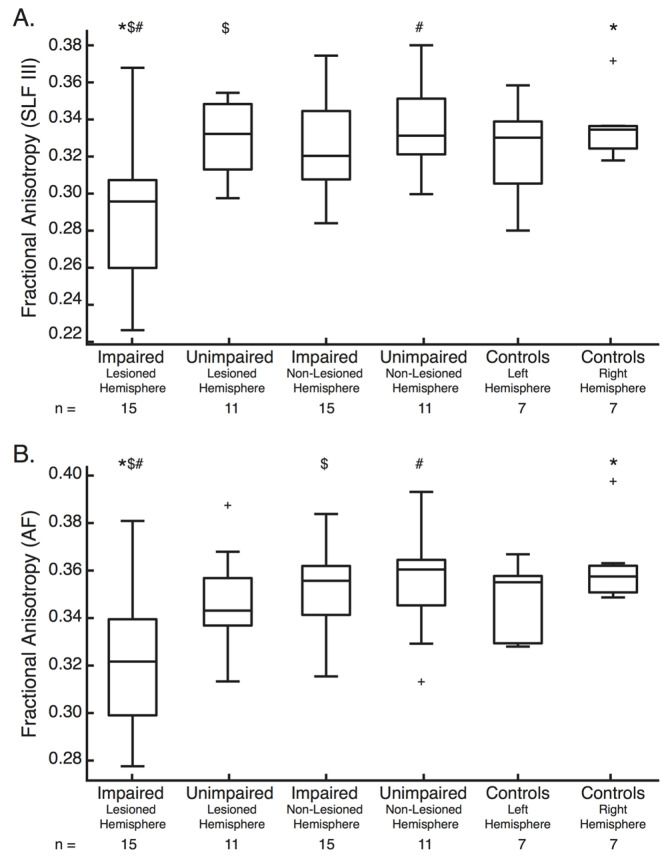
Fractional Anisotropy Box-plots—(**A**) Fractional anisotropy of the SLF III for the lesioned hemisphere of those with and without impairments on the Arm Position Matching Task, the non-lesioned hemisphere of those with (*n* = 15) and without (*n* = 11) impairments on the Arm Position Matching task and the left and right hemisphere of controls (*n* = 7). For each box, the centre line is the median and the bottom and top lines represent the 25th and 75th percentile, respectively. Whiskers extend to the highest and lowest data points, within 1.5 times the interquartile range from the top or bottom of the box. + indicate data points outside of this range and signify outliers. *, $ and # indicate the groups between which significant differences in Fractional anisotropy exist. (**B**) Same formats as (**A**), except for the AF. SLF = Superior Longitudinal Fasciculus, AF = Arcuate Fasciculus, FA = Fractional Anisotropy.

Differences in FA were assessed between those with impairments (*n* = 15) on the APM task (Task Score > or equal to 1.96), those without impairments (*n* = 11) on the APM task (Task Score < 1.96) and controls (*n* = 7). Significant effects were observed for the SLF III and AF only (Figure 5). There were no significant differences for the SLF I (F(5,60) = 1.21, *p* = 0.32), SLF II (F(5,60) = 2.02, *p* = 0.089) or MdLF (F(5,60) = 1.55, *p* = 0.19) (Supplemental Results SC). As such, post hoc analysis is only presented for the SLF III and AF (Figure 5). Across all tracts, FA was not significantly different between the left and right hemispheres of control participants. Those with impairments had lesions to many of the white matter tracts of interest. Those without impairments typically only had lesions impacting the AF, or did not have lesions impacting any of the tracts in question (Supplemental Results SB).

#### 3.4.1. SLF III

For the SLF III (F(5,60) = 4.10, *p* = 0.0029) (Figure 5a), FA was significantly lower in the lesioned hemisphere in those with impairments than both the lesioned and non-lesioned hemispheres of those without impairments (*p* = 0.024 and 0.0064 respectively). FA was also significantly lower in the lesioned hemisphere in those with impairments than the right hemisphere of controls (*p* = 0.025).

#### 3.4.2. AF

For the AF (F(5,60) = 4.02, *p* = 0.0033) (Figure 5b), FA was also significantly lower in the lesioned hemisphere of those with impairments than both the non-lesioned hemisphere of those with impairments (*p* = 0.024) and the non-lesioned hemisphere of those without impairments (*p* = 0.014). FA was also significantly lower in the lesioned hemisphere in those with impairments than the right hemisphere of controls (*p* = 0.0091).

### 3.5. Differences in MD and Tract Volume between Those Impaired and Those Unimpaired on the APM Task

A significant difference in MD, between those with impairments, those without impairments and controls, was observed for the SLF III only (F(5,60) = 3.288, *p* = 0.011) (Supplemental Results SA). This effect was driven by a significant difference between the lesioned hemisphere of those with impairments and the right hemisphere of controls (*p* = 0.012). No other significant differences were observed in MD for any other tract. The only significant difference in tract volume existed for the AF. Tract volume was lower in the lesioned hemisphere of those with impairments than the lesioned hemisphere of those without impairments (Supplemental Results SA).

### 3.6. Relationship between FA, MD, Tract Volume and APM Task Scores

**Figure 6:**
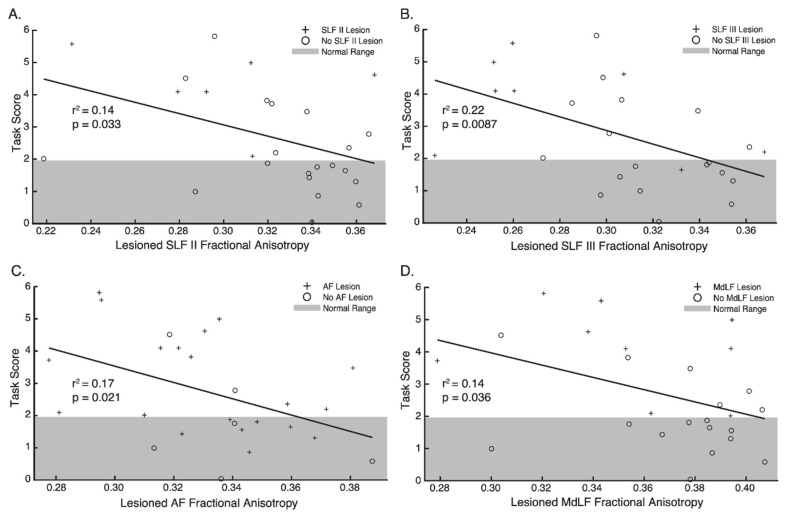
Regression plots—Relationship between Fractional Anisotropy and Arm Position Matching Task Scores for (**A**) SLF II, (**B**) SLF III, (**C**) AF and (**D**) MdLF. Crosses indicate participants with lesions impacting each tract respectively. Circles indicate participants without lesions impacting each tract respectively. Grey area denotes 95% of the normative range for age and sex matched controls. Points outside of this area indicate impairment on the Arm Position Matching task.

Analysis of the relationship between FA in the lesioned hemisphere and performance on the APM task revealed that FA was significantly associated with APM Task Scores for the SLF II, SLF III, AF and MdLF (Figure 6, Table 2). No statistically significant relationships were found between MD or Tract Volume and APM Task Scores (Table 2).

### 3.7. Classification of APM Task Impairments from Connectome and Diffusion Data

**Figure 7:**
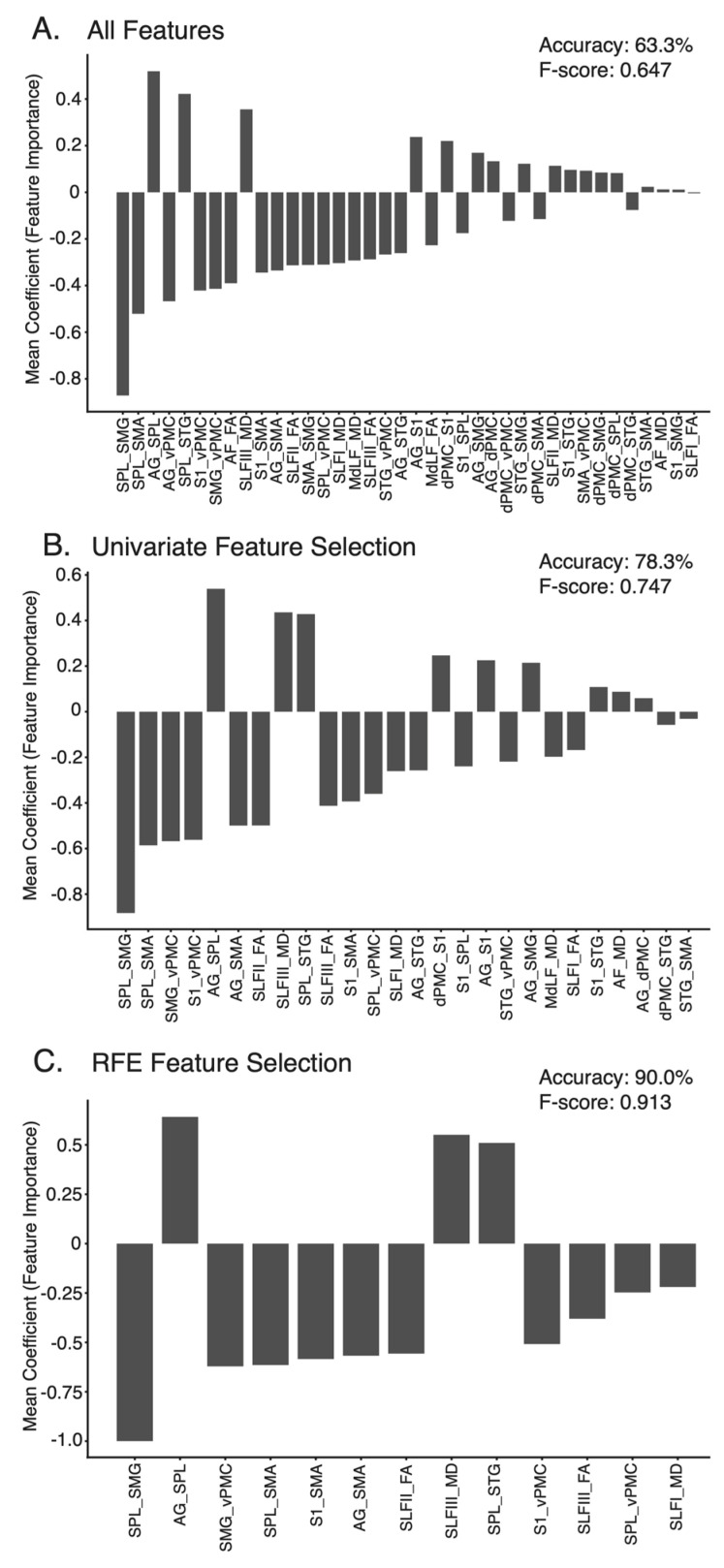
Feature Importance—Importance weighting of each feature in the (**A**) All Features model, (**B**) Univariate Feature Selection model, (**C**) Recursive Feature Elimination model across the ten cross-validation folds. Features ranked in order of absolute importance. SPL = Superior Parietal Lobule, SMG = Supramarginal gyrus, SMA = Supplementary Motor Area, AG = Angular gyrus, vPMC = Ventral Premotor Cortex, STG = Superior Temporal gyrus, S1 = Somatosensory Cortex, AF = Arcuate Fasciculus, FA = Fractional Anisotropy, SLF = Superior Longitudinal Fasciciuls, MD = Mean Diffusivity, MdLF = Middle Longitudinal Fasciculus, dPMC = Dorsal Premotor Cortex.

Table 3 displays the results for each of the three classification models. The All Features model was the least accurate model trained, accurately classifying impairment status 63.3% of the time (Table 3). The features, most important to classification in each model, are presented in Figure 7. For the All Features model, the top five most important features were connectivity between Superior Parietal Lobule and Supramarginal Gyrus (SPL–SMG), Angular Gyrus and Superior Parietal Lobule (AG–SPL), Superior Parietal Lobule and Supplementary Motor Area (SPL–SMA), Angular Gyrus and ventral Premotor Cortex (AG–vPMC) and S1 and ventral Premotor Cortex (S1–vPMC) (Figure 7a). The Univariate Feature Selection model accurately classified impairment status 78.3% of the time (Table 3). The top five features, carrying the most importance for this classification, were again connectivity between: SPL–SMG, SPL–SMA, S1–vPMC, Supramarginal Gyrus and ventral Premotor Cortex (SMG–vPMC) and AG–SPL. FA values for the SLF II and III were amongst the top 10 most important features (Figure 7b). Finally, the Recursive Feature Elimination model was, unsurprisingly, the most accurate model trained, accurately classifying impairment status 90.0% of the time (Table 3). Again, the top five most important features for this classification were connectivity between: SPL–SMG, AG–SPL, SMG–vPMC, SPL–SMA and S1 and Supplementary Motor Area (S1–SMA). FA values for the SLF II and SLF III were also amongst the features contributing to the Recursive Feature Elimination model (Figure 7c).

## 4. Discussion

This study highlights a clear importance for the SLF, AF and MdLF in proprioception, demonstrating that a number of white matter tracts within the brain subserve proprioception and contribute towards successful APM performance. When lesions impacted each of the SLF I, SLF II, SLF III, AF and MdLF, performance was worse than that of controls.

Interestingly, differences in FA were less apparent between those with and without APM task impairments, except for the SLF III. Despite this, significant associations were still observed between FA in the lesioned hemisphere and APM Task Scores for the SLF II, SLF III, AF and MdLF (Table 2, Figure 6). Finally, structural connectivity measures, surrounding a network of regions connected by the SLF, AF and MdLF contributed to accurate classification of impairment status on the APM task following stroke (Table 3, Figure 7).

### 4.1. The Superior Longitudinal Fasciculus in Human Proprioception

Lesions to each branch of the SLF (Figure 4) and decreases in FA for the SLF II and SLF III were associated with worse APM Task performance (Figure 6, Table 2). Furthermore, the SLF III was the only white matter tract where FA was found to be lower in the lesioned hemisphere of those with impairments than those without impairments (Figure 5a).

Most evidence supporting a proprioceptive function of the SLF exists for the cortical regions it connects. Functional neuroimaging work in humans shows inferior parietal lobe (particularly the supramarginal gyrus) and premotor cortex involvement during tasks including: passive movement [14,22,87,88], vibratory illusions [11,19,21,87,89,90,91], tasks requiring precision vs. power grip [92], the remapping of visual stimuli to changes in limb position [93], encoding sensory information in body-centered coordinates [94], as well as position matching tasks, as used in the current study [15,23].

Interestingly, greater position matching errors in stroke participants have been associated with decreased activation in regions connected through the SLF III; in the supramarginal gyrus during the passive arm movements of the task, and in the premotor cortex during the active matching component of the task [15]. These position matching studies also highlighted activation in regions likely connected by the SLF I [95]; the supplementary motor area and the superior parietal lobe [15,23].

While the aforementioned studies demonstrated proprioceptive activity in regions surrounding the SLF, the current study demonstrated the direct importance of the SLF connections for successful performance in an APM task. It is likely the SLF, connecting the parietal and frontal lobes, is critical in the communication between these cortical regions when formulating the spatial representations of the upper limb, necessary to accurately perform the APM task. This postulate is in line with work in non-human primates, suggesting that these intraparietal-premotor projections are responsible for the spatial encoding of movement [96]. As such, lesions to the SLF, and decreases in FA which potentially disrupt communication between the parietal and frontal lobes, could cause the difficulties in APM task performance observed in stroke participants in this study.

### 4.2. The Arcuate Fasciculus in Imitation

Like the SLF, worse APM task performance was associated with lesions and changes in FA for the AF (Figure 4, Figure 5 and Figure 6 and Table 2). The vast majority of literature supports the role of the left AF in language [35,38,45,97]. While the AF’s role in language seems undisputed, the current study is not the first to associate the AF with proprioception, with lesions impacting the AF having previously been associated with proprioceptive impairments both one- and six-months post stroke [18]. Other work has suggested a role for the AF in visual and proprioceptive imitation and the transformations required to turn perception into action [98,99]. In many ways, the APM task could be considered an imitation task. Participants were required to perceive the location of the affected limb, generate the appropriate spatial transformation, and imitate the position with the unaffected limb, thus implicating the AF.

### 4.3. The Middle Longitudinal Fasciculus, Superior Longitudinal Fasciculus and Attention

Lesions to the MdLF and decreases in FA of the MdLF were both significantly associated with worse APM task performance (Figure 4 and Figure 6, Table 2). The MdLF has connections between superior temporal regions and parietal regions, including the SPL, AG and SMG [51,52,56]. All of these cortical regions have been implicated in spatial attention [51,100,101,102,103] and proprioception [15,16,17,19,21]. Additionally, many white matter investigations have also implicated the SLF and MdLF with neglect [101,103,104,105,106], while others have suggested the importance of the MdLF in language processing, through presumed changes in attentional biases [107].

It has been suggested that lesions to the regions interconnected by the MdLF and SLF, and reduced activation of these regions, could result in poorer performance on APM tasks due to reduced attention to the spatial location of the limb [17,18,23,57]. Although the majority of stroke participants (24/26) in the present study did not have neglect, clinically, it is suggested that pen and paper tests might not detect those with mild attentional deficits while performing more complex, functional tasks [58]. Given the extensive involvement of the grey and white matter involvement of both the MdLF and SLF in neglect, it would be inappropriate to not give recognition to the idea that the poor performance seen in some participants could be due to mild inattention to the spatial state of the upper limb.

### 4.4. Proprioceptive Network Connectivity Predicts Proprioceptive Impairment

Structural connectivity measures surrounding the cortical regions connected by the SLF, AF and MdLF were able to accurately predict whether participants would be impaired on the APM task (Table 3). It was unsurprising that accuracy and all other performance metrics improved in models utilizing feature selection techniques. This was presumably as noisy features containing little predictive utility were reduced in the dataset.

FA is commonly used as a metric when studying white matter and neurological conditions in the human brain. The SLF II and SLF III were the only tracts where FA had relatively high predictive value in each model. While FA and MD of these long association fibres were useful features in each predictive model, particularly for the SLF branches, connectivity values between other regions offered more importance in each model. This was particularly true for the connectivity between the superior parietal lobe, supramarginal gyrus and angular gyrus (Figure 7c), regions known to be connected via short association fibres [25], but also between parietal and frontal regions (e.g., SMG–vPMC, SPL–SMA, S1–SMA, AG–SMA, S1–vPMC and AG–vPMC). Although the predominant focus of the present manuscript was on the long association fibres connecting parietal, frontal and temporal regions, it is crucial not to overlook the importance of short white matter tracts connecting adjacent cortical areas, when understanding proprioceptive processing at a network level. Connectivity metrics other than FA, such as the number of streamlines connecting regions of the brain, might provide additional neuroanatomical insight as to the connectivity between cortical regions responsible for proprioceptive tasks.

Previous work has demonstrated a wide cortical network responsible for accurate proprioception after stroke [16,17,57]. Given the findings in the current study, it is likely that a similar intricate network of white matter tracts, connecting these cortical regions through both long and short association fibres, is equally important for supporting accurate proprioception following stroke.

### 4.5. Limitations

Like any other study, the current work unfortunately comes with its limitations. While the lateralization of function is of interest, the sample size in this study did not allow for this distinction to be made for each of the white matter tracts. This is particularly unfortunate for the arcuate fasciculus, where the literature is dominated by implications of the left hemisphere in language. Future considerations should be made for the involvement of the respective left and right hemisphere tracts in proprioception.

As with all behavioural studies taking place during the early sub-acute stage post-stroke, it is always a possibility that other factors such as fatigue and cognitive deficits may influence stroke participants true performance on these complex behavioural tasks. While every attempt is made to reduce the impact of fatigue, with the APM task being relatively short and cognitively, stroke participants were capable of performing the APM task, it is still fair to acknowledge that these factors may compound participant performance on the task.

Unfortunately, the lesions observed in the study sample were often not mutually exclusive to a single white matter tract (Supplemental Results SB). Stroke affects vascular territories rather than exclusive brain regions and many of the white matter tracts of interest run parallel with one other. It is plausible that the impact of multiple white matter tracts contributes to the deficits observed in the APM task, in ways that are difficult to parcellate using naturally occurring strokes, or without large sample sizes.

### 4.6. Clinical Significance

Proprioception is commonly affected following stroke; however, clinical testing of proprioception, without the use of instrumented methods, lacks sensitivity to detect subtle impairments [76,108,109]. Improving the understanding of the neural correlates of proprioceptive impairment will lead to better clinical diagnosis of these impairments, through greater awareness. As such, a more complete understanding the neural correlates will lead to more personalized rehabilitation and better outcomes for these individuals in the future.

## 5. Conclusions

This study demonstrated how major cortico–cortical white matter connections, particularly the SLF and AF, are critical for proprioception following stroke. It advances our understanding of the white matter correlates of proprioception within the brain, extending beyond the commonly discussed DCML pathway. Future studies should continue to investigate the role of the SLF, AF and MdLF in proprioception, considering the potential for lateralization of proprioceptive function within them.

## Figures and Tables

**Table 1:** Demographics—Demographic information for stroke and control participants. TLT = Thumb Localisation Test, BIT = Behavioural Inattention Test, FIM = Functional Independence Measure, CMSA = Chedoke McMaster Stroke Assessment, MoCA = Montreal Cognitive Assessment.

	Stroke	Controls
**Age (mean ± sd)**	63.0 ± 13.4	62.1 ± 6.2
**Sex n[M,F]**	[16, 10]	[3, 4]
**Handedness n[L,A,R]**	[0, 1, 25]	[0, 0, 7]
**Lesioned Hemisphere n[L,R]**	[7, 19]	N/A
**Affected Arm n[L,R]**	[19, 7]	N/A
**Lesion Volume (mean ± sd)**	11.97 cc ± 16.60 cc	N/A
**BIT (median:range)**	143: 100–146	N/A
FIM (median: range) *	101: 70–126	N/A
**CMSA affected arm n [1,2,3,4,5,6,7]**	[0, 3, 4, 3, 6, 5, 5]	N/A
**CMSA unaffected arm n [1,2,3,4,5,6,7]**	[0, 0, 0, 0, 1, 3, 22]	N/A
**MoCA (median: range) ^$^**	25: 15–30	N/A
**Days from Stroke to Imaging (mean ± sd)**	27.1 ± 11.2	N/A
**Days from Stroke to Clinical (mean ± sd)**	21.3 ± 11.0	N/A
**Days from Stroke to Robot time (mean ± sd)**	22.6 ± 11.9	N/A

* No FIM scores for 1 participant; ^$^ MoCA scores not available for 2 participants.

**Table 2:** Linear Regression Results—Metrics of the regression analysis between Fractional Anisotropy, Mean Diffusivity and Tract Volume and Arm Position Matching Task Scores for each white matter tract. *p*-values in bold indicate those which are significant at the respective q-value. SLF = Superior Longitudinal Fasciculus, AF = Arcuate Fasciculus, MdLF = Middle Longitudinal Fasciculus, FA = Fractional Anisotropy, MD = Mean Diffusivity.

	Intercept	Coefficient	*p*-Value
**SLF I**			
FA	4.96	−6.61	0.52
MD	3.31	−691.86	0.90
Tract Volume	3.46	−0.067 × 10^−3^	0.35
**SLF II**			
FA	8.31	−17.50	**0.033**
MD	−3.13	6573.40	0.26
Tract Volume	2.54	0.015 × 10^−3^	0.76
**SLF III**			
FA	9.22	−21.15	**0.0087**
MD	−5.86	9171.67	0.078
Tract Volume	2.99	−0.024 × 10^−3^	0.74
**AF**			
FA	11.11	−25.27	**0.021**
MD	−1.99	5444.11	0.42
Tract Volume	4.12	−0.034 × 10^−3^	0.18
**MdLF**			
FA	9.68	−19.03	**0.036**
MD	2.70	−6.81	1.00
Tract Volume	5.61	−0.012 × 10^−2^	0.14

**Table 3:** Logistic Regression Classification—Accuracy, Precision, Recall and F1-Score for the All Features model, Univariate Feature Selection model and Recursive Feature Elimination model. # of Features Included = Number of Features Included.

Model	Accuracy	Precision	Recall	F1-Score	# of Features Included
Logistic Regression					
**All Features model (10-fold)**	63.3%	76.7%	73.3%	0.647	38
**Univariate Feature Selection model (10-fold)**	78.3%	86.7%	80%	0.747	26
**Recursive Feature Elimination model (10-fold)**	90.0%	86.7%	100%	0.913	13

## Data Availability

The data presented in this study is not publicly available due to restrictions on data sharing without formal data sharing agreements in place, as determined by the Institutional Review Board. Those wishing to obtain the study data should contact the corresponding author (S.P.Dukelow).

## Supplementary Materials

The following supporting information can be downloaded at: https://www.mdpi.com/article/10.3390/brainsci12121651/s1. Supplemental Material: Supplemental Methods SA, description of robotic parameters and their calculations; Supplemental Results SA, mean diffusivity and tract volume for stroke and control participants; Supplemental Results SB, a list of white matter tracts lesions for each participant; Supplemental Results SC, fractional anisotropy for the SLF I, SLF II and MdLF. References [76,77] are cited in Supplemental Methods SA.
