# Supplementary material for: Beyond the Dorsal Column Medial Lemniscus in Proprioception and Stroke: A White Matter Investigation
Source: Brain Sci. 2022 Dec 2;12(12):1651. doi: 10.3390/brainsci12121651 (PMC9775186; doi:10.3390/brainsci12121651)
Supplement: Supplementary file 1 [file brainsci-12-01651-s001.zip › brainsci-1996337-supplementary.pdf]

Supplemental Methods SA

APM Task parameter calculations

Absolute error was simply calculated as the mean absolute distance error across all trials of the APM task, in the x, y and combined xy directions. *Variability* refers to the consistency in ability to match arm position. It is calculated by taking the mean of the standard deviations for the participants hand positions for both the x and y directions, for each of the nine targets. *VariabilityXY* was then calculated by taking the root sum-square (RSS) of the variabilities in the x and y directions. *Contraction/expansion* refers to the area of the workspace matched by the active arm relative to that of the passive arm. *Contraction/expansion*, in the x and y directions, was calculated by taking the absolute difference between the mean positions of the three left-most targets and the three right-most targets (proximal and distal targets for the y direction) of the active arm and dividing it by the same range of movement for the passive arm. *Contraction/expansionXY* was calculated by calculating the ratio of the area created by the mean positions of the eight outer-most targets for the active and passive arms respectively. *Shift* describes a systematic shift in the perceived arm-position in the x or y direction. It was calculated as the mean difference between the mirrored x and y positions of the active arm and those of the passive arm. Positive values are for lateral and distal shifts and negative values for medial and proximal shifts. *ShiftXY* was calculated as the RSS of the shifts in x and y respectively. Details on each of these parameters have previously been published elsewhere [76,77].

Supplemental Results SA—Average MD and tract volume for each of the SLF I, SLF II, SLF III, AF and MD across each hemisphere of control participants (n = 7) and those with (n = 15) and without (n = 11) APM task impairments. Values represent the mean ± standard deviation. [a] and [b] indicate group comparisons where significant differences exist.

	MD (mm^2/s)	Tract Volume (cc)
**SLF I**		
Left Controls	0.00090 ± 8.25 $x10^{-5}$	12.68 ± 4.05
Right Controls	0.00091 ± 9.15 $x10^{-5}$	11.08 ± 1.72
Impaired (Lesioned Hemisphere)	0.00088 ± 5.04 $x10^{-5}$	10.10 ± 4.15
Impaired (Non-Lesioned Hemisphere)	0.00087 ± 5.25 $x10^{-5}$	9.65 ± 5.06
Unimpaired (Lesioned Hemisphere)	0.00089 ± 7.30 $x10^{-5}$	13.17 ± 4.70
Unimpaired (Non-Lesioned Hemisphere)	0.00086 ± 6.48 $x10^{-5}$	12.19 ± 6.62
**SLF II**		
Left Controls	0.00088 ± 3.81 $x10^{-5}$	12.60 ± 3.55
Right Controls	0.00086 ± 4.47 $x10^{-5}$	9.50 ± 2.96
Impaired (Lesioned Hemisphere)	0.00090 ± 5.05 $x10^{-5}$	9.35 ± 5.70
Impaired (Non-Lesioned Hemisphere)	0.00090 ± 6.69 $x10^{-5}$	11.59 ± 3.58
Unimpaired (Lesioned Hemisphere)	0.00087 ± 6.19 $x10^{-5}$	10.60 ± 7.94
Unimpaired (Non-Lesioned Hemisphere)	0.00087 ± 7.60 $x10^{-5}$	9.19 ± 4.27
**SLF III**		
Left Controls	0.00090 ± 6.14 $x10^{-5}$	13.58 ± 2.86
Right Controls	0.00086 ± 5.73 $x10^{-5}$ [a]	14.73 ± 2.05
Impaired (Lesioned Hemisphere)	0.00095 ± 6.10 $x10^{-5}$ [a]	11.29 ± 5.39
Impaired (Non-Lesioned Hemisphere)	0.00092 ± 5.40 $x10^{-5}$	13.68 ± 3.97
Unimpaired (Lesioned Hemisphere)	0.00091 ± 5.26 $x10^{-5}$	13.80 ± 2.70
Unimpaired (Non-Lesioned Hemisphere)	0.00089 ± 5.07 $x10^{-5}$	14.91 ± 3.87
**AF**		
Left Controls	0.00084 ± 2.31 $x10^{-5}$	41.77 ± 14.41
Right Controls	0.00081 ± 3.21 $x10^{-5}$	47.61 ± 11.91
Impaired (Lesioned Hemisphere)	0.00086 ± 5.36 $x10^{-5}$	36.28 ± 10.82[b]
Impaired (Non-Lesioned Hemisphere)	0.00084 ± 4.14 $x10^{-5}$	41.47 ± 9.68

Unimpaired (Lesioned Hemisphere)	$0.00086 \pm 4.23 \times 10^{-5}$	50.00 ± 11.04[b]
Unimpaired (Non-Lesioned Hemisphere)	$0.00085 \pm 5.67 \times 10^{-5}$	42.84 ± 10.92
**MdLF**		
Left Controls	$0.00082 \pm 4.16 \times 10^{-5}$	24.38 ± 6.37
Right Controls	$0.00081 \pm 3.39 \times 10^{-5}$	25.75 ± 3.56
Impaired (Lesioned Hemisphere)	$0.00084 \pm 6.53 \times 10^{-5}$	23.83 ± 4.28
Impaired (Non-Lesioned Hemisphere)	$0.00082 \pm 4.46 \times 10^{-5}$	23.62 ± 4.25
Unimpaired (Lesioned Hemisphere)	$0.00085 \pm 6.47 \times 10^{-5}$	26.78 ± 3.46
Unimpaired (Non-Lesioned Hemisphere)	$0.00085 \pm 7.52 \times 10^{-5}$	26.82 ± 3.68

Supplemental Results SB—Identification of the white matter tracts that were lesioned for each participant. ✓indicates a lesion to that respective white matter tract. X indicates no lesion to that respective white matter tract. Arm Position Matching (APM) Task Scores for individual participants are also presented. APM Task Scores that are in bold indicate those participants with APM Task impairments.

**Participant**	**SLF I Lesion**	**SLF II Lesion**	**SLF III Lesion**	**AF Lesion**	**MdLF Lesion**	**APM Task Score**
01	X	X	X	X	X	0.58
02	X	X	X	✓	X	1.56
03	X	✓	✓	✓	✓	**2.09**
04	X	X	X	✓	X	**2.36**
05	X	X	X	X	X	0.04
06	X	X	X	✓	X	1.31
07	✓	✓	✓	✓	✓	**4.99**
08	X	X	X	X	X	0.99
09	X	X	X	✓	X	1.87
10	X	X	✓	✓	X	**2.20**
11	X	X	X	X	X	1.76
12	X	X	X	✓	✓	**2.02**
13	X	X	X	✓	X	**3.48**
14	X	X	X	X	X	**4.51**
15	X	X	X	✓	X	**3.82**
16	X	X	X	✓	✓	**5.81**
17	X	X	X	✓	✓	**3.72**
18	X	X	✓	✓	X	1.65
19	X	X	X	X	X	**2.78**
20	X	X	X	✓	X	0.86
21	X	✓	✓	✓	✓	**4.62**
22	X	✓	✓	✓	✓	**4.10**
23	X	X	X	✓	X	1.43
24	✓	✓	✓	✓	✓	**4.10**
25	X	X	X	✓	X	1.81
26	✓	✓	✓	✓	✓	**5.58**

Supplemental Results SC—Fractional Anisotropy Box-plots – A) Fractional anisotropy of the SLF I for the lesioned hemisphere of those with and without impairments on the Arm Position Matching Task, the non-lesioned hemisphere of those with (n = 15) and without (n = 11) impairments on the Arm Position Matching task and the left and right hemisphere of controls (n = 7). [*], [$] and [#] indicate the groups between which significant differences in Fractional anisotropy

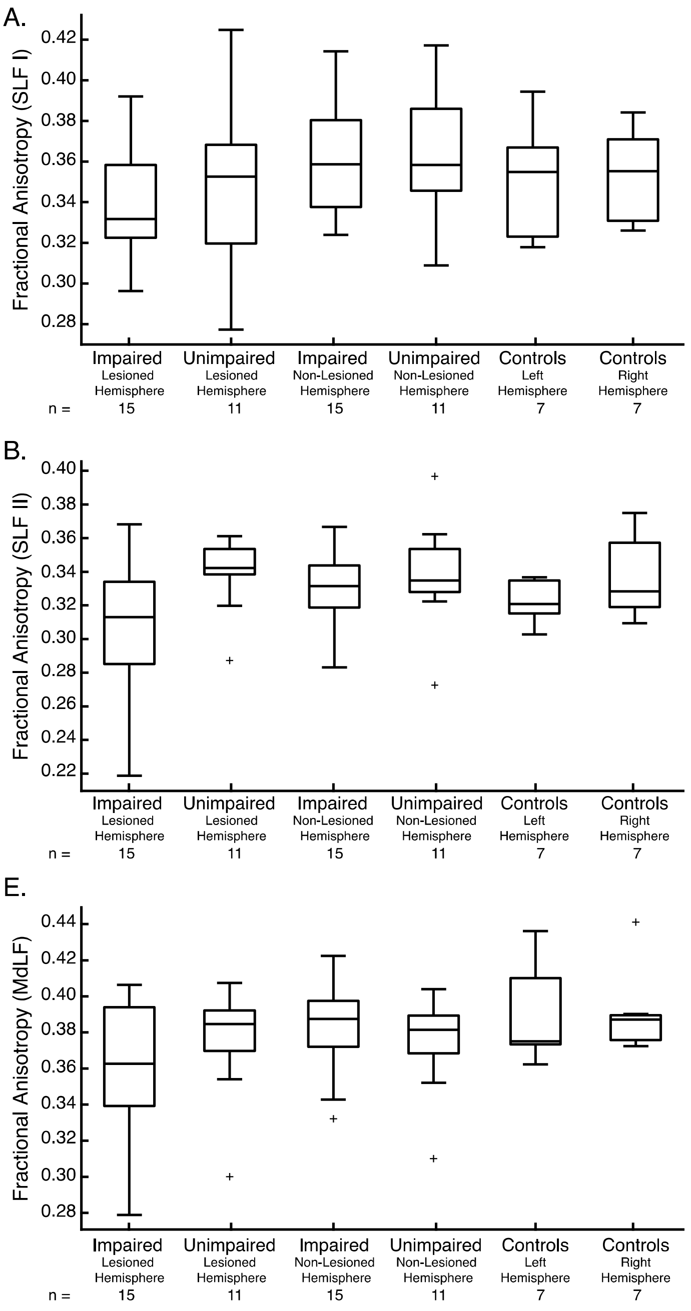

exist. + indicate outliers. B and C) Same formats as A, except for the SLF II and MdLF. SLF = Superior Longitudinal Fasciculus, MdLF = Middle Longitudinal Fasciculus, FA = Fractional Anisotropy.
